# Supplementary material for: Untargeted lipidomic analysis of metabolic dysfunction-associated steatohepatitis in women with morbid obesity
Source: PLoS One. 2025 Mar 4;20(3):e0318557. doi: 10.1371/journal.pone.0318557 (PMC11878907; doi:10.1371/journal.pone.0318557)
Supplement: Attachment — Submitted filename: Response to Reviewers.docx [file pone.0318557.s007.docx]

**Response to Reviewers**

Review Comments to the Author:

**Reviewer #1:** The manuscript titled "Untargeted Lipidomic Analysis of Metabolic Dysfunction-Associated Steatohepatitis Associated with Morbid Obesity" presents a significant contribution to the study of Metabolic Dysfunction-Associated Steatohepatitis (MASH) in the context of morbid obesity. The authors conducted an untargeted lipidomic analysis using LC/MS on serum samples from 216 women, identifying lipid profiles across different histological stages of Metabolic Dysfunction-Associated Steatotic Liver Disease (MASLD). The findings provide valuable insights into potential non-invasive biomarkers for MASH.

While the manuscript is well-structured and addresses an important gap in the literature, there are several areas that require improvement to enhance clarity, methodological transparency, and overall impact. Below are major and minor revisions to consider.

Major Corrections:

- The introduction would benefit from a more comprehensive discussion of the key lipid metabolites implicated in MASH and MASLD, with reference to current literature. By providing this background, readers will better understand the study's significance and how it builds on or diverges from previous work.

**Response:** Thank you for this suggestion. We have revised the introduction to include a more comprehensive review of the lipid metabolites associated with MASH and MASLD. The updated text now incorporates additional references [14-16] and a contextual discussion on ceramides, triacylglycerols, phospholipids, and their roles in MASLD progression and MASH pathogenesis.

- The manuscript references previous work regarding LC/MS methods, but a more detailed explanation of cleaning data, quality controls, preprocessing steps (transformation, normalization, scaling and handling missing data) and software used, is needed. Ensuring full methodological transparency is critical for the reproducibility of the findings.

**Response:** We have expanded the Materials and Methods section to include a thorough explanation of our data preprocessing steps, including data cleaning, quality control measures, normalization, and scaling techniques. The specific software tools and packages used in the preprocessing have also been identified to ensure full methodological transparency.

- Table 1 would be more informative if global p-values and specific p-values were included for specific comparisons, along with statistical significance notation (e.g., * for p < 0.05, ** for p < 0.01, *** for p < 0.005). This would make the statistical relevance of the data clearer and easier to interpret.

**Response:** We did not consider it appropriate to add global p-values among the three groups, as they may lead to confusion and do not provide relevant significance, potentially confusing the reader. However, we have updated Table 1 to include specific comparisons across groups, with appropriate statistical notation (* for p < 0.05, ** for p < 0.01, and *** for p < 0.005). This addition enhances the interpretability and statistical relevance of the findings.

- Since Tables 2, 3, and 4 focus, it would be beneficial to merge them with the S1 Table, where p-values and fold changes are already provided. This consolidation will streamline data presentation, making it easier for readers to access all relevant information in one location.

**Response:** We agree that consolidating these tables would facilitate a more comprehensive view of the lipidomic data. Thus, we have merged the tables as suggested to include family names, p-values, fold changes, and statistical significance indicators for all groups in new S2, S3, S4 and S5 Tables. Consolidated tables will provide a more accessible resource for readers.

- Consider performing Partial Least Squares Discriminant Analysis (PLS-DA) to improve the discriminatory power between the different experimental groups of the study. Furthermore, the best biomarkers could be evaluated in sensitivity analyses that account for potential confounders, such as alcohol consumption and comorbidities, would strengthen the study’s conclusions.

**Response:** We have generated a PLS-DA with all the variables and another one with the significant ones, but it does not show separation between the groups. We believe the model is weak, so we decided to perform a PCA with the significant variables, which we have added as the new Fig 1. Since there is also no clear separation between the groups, we cannot conduct a sensitivity analysis and have postponed this for future studies when this analysis can be performed with more concrete results. This was a preliminary study.

- Adding a Venn diagram or other visual representation of overlapping lipid metabolites across different experimental groups (e.g., NL, SS, MASH) would clarify the common and distinct lipid profiles. This would help visualize key findings and their relationships more effectively.

**Response:** A Venn diagram illustrating overlapping and distinct lipid profiles among NL, SS, and MASH groups has been added to the Results section as Fig 2, as you recommended.

- A critical issue is the lack of a clear Data Availability Statement in the current manuscript draft. According to PLOS ONE guidelines, data availability is a requirement for publication. The authors should ensure that they provide a detailed data availability section that explains how the underlying data can be accessed, either through a repository link or within the manuscript itself.

**Response:** We have added a Data Availability Statement as per PLOS ONE’s requirements, detailing how the data supporting our findings can be accessed.

Minor Corrections:

- The title should clearly reflect that the study was conducted exclusively on women. A more accurate title could be: "Untargeted Lipidomic Analysis of Metabolic Dysfunction-Associated Steatohepatitis in Women with Morbid Obesity".

**Response:** To clarify the study’s focus on a female cohort, we revised the title to: “Untargeted Lipidomic Analysis of Metabolic Dysfunction-Associated Steatohepatitis in Women with Morbid Obesity.”

- It is recommended to include the geographical details of the cohort, such as the hospital, city, and country, to provide context and ensure the generalizability of the findings.

**Response:** We added the specific location details of the cohort in the Materials and Methods section to provide context regarding generalizability.

- Including a correlation plot between clinical variables (e.g., BMI, glucose levels) and metabolites would offer deeper insights into the relationships within the data. A Principal Component Analysis (PCA) plot to visualize group separation based on lipidomic profiles would also be a useful addition.

**Response:** Given your advice, we have conducted multiple linear regressions with significant lipid metabolites against clinical variables, but the R^2 values do not exceed 0.25, indicating that the regressions are poor and not worth including (I attach below). This is better, as it suggests that the trends of these lipids are not influenced by these variables. However, we have included a PCA plot showing the separation of variables between the groups in the new Fig 1.

| **Qidx (LIPID METABOLITE)** | **R2** | **Glc** | **Hb1c** | **GOT** | **GPT** | **GGT** | **ALP** | **TGL** | **HDL** | **LDL** |
| --- | --- | --- | --- | --- | --- | --- | --- | --- | --- | --- |
| 31 | 0,096249907 | 0,039433545 | 0,924000069 | 0,352985232 | 0,225470022 | 0,611395986 | 0,465665863 | 0,000333216 | 0,598124431 | 0,000244818 |
| 70 | 0,227777773 | 0,033855871 | 0,9472855 | 0,024893097 | 0,28311685 | 0,017007716 | 0,277032273 | 0,004623627 | 0,000820325 | 0,033855871 |
| 84 | 0,185352099 | 0,027986496 | 0,403620691 | 0,025643765 | 0,092222605 | 0,190732424 | 0,511148494 | 0,383186173 | 0,284906028 | 0,9472855 |
| 97 | 0,10666593 | 0,113731659 | 0,641301995 | 0,801514504 | 0,804470677 | 0,301417208 | 0,94749029 | 0,764025352 | 0,002082254 | 0,024893097 |
| 100 | 0,186991762 | 0,038182289 | 0,377564242 | 0,114305077 | 0,236890018 | 0,031184086 | 0,32177079 | 0,822086394 | 0,012106039 | 0,28311685 |
| 102 | 0,228838717 | 0,001318485 | 0,98215037 | 0,029232697 | 0,073827203 | 0,019852382 | 0,147456463 | 0,14223533 | 0,004404966 | 0,017007716 |
| 103 | 0,231837868 | 0,031506214 | 0,919284863 | 0,022241387 | 0,233749599 | 0,010268541 | 0,231865671 | 0,021228374 | 0,138740618 | 0,277032273 |
| 110 | 0,082074017 | 0,072470231 | 0,01380248 | 0,075296042 | 0,060365043 | 0,306944862 | 0,8101954 | 0,003264979 | 0,150080874 | 0,004623627 |
| 129 | 0,185968416 | 0,058983091 | 0,011092459 | 0,00468361 | 0,186980386 | 0,552191641 | 0,2745534 | 0,115580908 | 0,005961161 | 0,188882687 |
| 149 | 0,237332693 | 0,001479181 | 0,311740841 | 0,025795801 | 0,221437937 | 0,018301213 | 0,32011523 | 0,002488531 | 0,129863865 | 1,92556E-06 |
| 173 | 0,072819547 | 0,455703616 | 0,065020681 | 0,416571035 | 0,946451278 | 0,848620394 | 0,313190857 | 0,011653511 | 0,343832058 | 3,75138E-05 |
| 194 | 0,241329784 | 2,56056E-05 | 0,022682089 | 0,017765124 | 0,092584189 | 0,020003059 | 0,192858538 | 0,033508145 | 0,01380248 | 0,115321997 |
| 194 | 0,114538892 | 0,003857092 | 0,418407356 | 0,080241181 | 0,028740887 | 0,764405085 | 0,884426637 | 0,166018528 | 0,010296984 | 1,48946E-05 |
| 222 | 0,147583864 | 0,001220319 | 0,630379096 | 0,101666252 | 0,038079539 | 0,598124431 | 0,726391795 | 0,019411648 | 0,003279927 | 0,012429162 |
| 236 | 0,169706805 | 0,03271189 | 0,870255501 | 0,600022501 | 0,813748411 | 0,005961161 | 0,399657603 | 0,170340198 | 0,132429496 | 0,048668941 |
| 237 | 0,166831339 | 0,000143623 | 0,235879005 | 0,060169877 | 0,023087197 | 0,381910586 | 0,448628438 | 0,089998628 | 0,075296042 | 0,080998999 |
| 240 | 0,212753192 | 7,23705E-06 | 0,003264979 | 0,011653511 | 0,033508145 | 0,089998628 | 0,165224593 | 0,012429162 | 0,060365043 | 0,234020893 |
| 253 | 0,170141963 | 0,000224026 | 0,138740618 | 0,150080874 | 0,343832058 | 0,072833636 | 0,037887112 | 0,048668941 | 0,306944862 | 0,08059216 |
| 257 | 0,169519962 | 0,002512233 | 0,890190292 | 0,046531012 | 0,03805222 | 0,129863865 | 0,373765181 | 0,015322666 | 0,018769645 | 0,066821092 |
| 258 | 0,22542776 | 2,00549E-05 | 0,04554025 | 0,019539867 | 0,079917556 | 0,03144054 | 0,026200453 | 0,066821092 | 0,8101954 | 0,012495746 |
| 284 | 0,218192218 | 0,012973004 | 0,565779333 | 0,383179494 | 0,523486017 | 0,010296984 | 0,725407442 | 0,813748411 | 0,115561381 | 0,03668759 |
| 289 | 0,222268772 | 0,005013217 | 0,819343325 | 0,355803514 | 0,62967492 | 0,003279927 | 0,486779387 | 0,03805222 | 0,139151398 | 0,000820325 |
| 300 | 0,206725343 | 6,12866E-06 | 0,012033244 | 0,021165773 | 0,037798076 | 0,132429496 | 0,209268744 | 0,523486017 | 0,006442307 | 0,002507264 |
| 305 | 0,264187619 | 4,33787E-05 | 0,000333216 | 0,001434219 | 0,023699219 | 0,018769645 | 0,240049961 | 0,62967492 | 0,021228374 | 0,001193454 |
| 308 | 0,174560686 | 0,000150954 | 0,110017935 | 0,007370346 | 0,02459448 | 0,348124134 | 0,08167932 | 0,03668759 | 0,000244818 | 0,035312097 |
| 321 | 0,222085955 | 3,91935E-05 | 0,023893801 | 0,006838595 | 0,018829019 | 0,046030733 | 0,366227432 | 0,127611965 | 0,127373681 | 0,537645224 |
| 357 | 0,253224754 | 3,3994E-06 | 0,022725772 | 0,005143546 | 0,019480192 | 0,025805265 | 0,038187983 | 0,001193454 | 0,195514081 | 0,036060644 |
| 364 | 0,211702346 | 8,62556E-05 | 0,115580908 | 0,00563176 | 0,008445586 | 0,115561381 | 0,528598836 | 0,037798076 | 0,019539867 | 0,004466899 |
| 386 | 0,22514408 | 0,002035146 | 0,002488531 | 0,024970749 | 0,376356287 | 0,030400354 | 0,222886862 | 0,023699219 | 0,007370346 | 0,000652392 |
| 400 | 0,231113785 | 0,002899494 | 0,166018528 | 0,008102579 | 0,176500775 | 0,019334069 | 0,012331319 | 0,008445586 | 0,006838595 | 0,004623627 |
| 435 | 0,108540723 | 0,319421743 | 0,019411648 | 0,197978672 | 0,701319698 | 0,722102 | 0,129900241 | 0,376356287 | 0,005143546 | 0,000807277 |
| 455 | 0,181257745 | 4,90176E-05 | 0,170340198 | 0,084577433 | 0,139151398 | 0,190773778 | 0,056339754 | 0,115580908 | 0,195514081 | 0,035312097 |
| 467 | 0,178995648 | 0,000244818 | 0,127373681 | 0,195514081 | 0,472475646 | 0,036517553 | 0,018581344 | 0,275717494 | 0,472475646 | 0,167428774 |
| 481 | 0,168142158 | 0,188882687 | 0,21736716 | 0,621958828 | 0,354654967 | 0,007178249 | 0,112871203 | 0,343568239 | 0,036517553 | 0,00095621 |
| 486 | 0,242008023 | 1,92556E-06 | 0,007739967 | 0,010061166 | 0,024220143 | 0,030030831 | 0,059975741 | 0,409033904 | 0,030030831 | 0,055152107 |
| 501 | 0,257574535 | 1,48946E-05 | 0,027390614 | 0,001229325 | 0,007438933 | 0,017900116 | 0,056884162 | 0,102062501 | 0,25945317 | 0,003441326 |
| 547 | 0,115575294 | 0,271534622 | 0,015322666 | 0,188596873 | 0,716318259 | 0,995375858 | 0,210210502 | 0,002488531 | 0,621958828 | 4,69595E-05 |
| 559 | 0,119627982 | 0,265336227 | 0,013735909 | 0,139842403 | 0,551594664 | 0,813702593 | 0,112056739 | 0,166018528 | 0,010061166 | 0,121068547 |
| 560 | 0,117941938 | 0,205851746 | 0,010672726 | 0,158251059 | 0,554697301 | 0,807279046 | 0,11214212 | 0,019411648 | 0,001229325 | 0,003441326 |
| 599 | 0,165444286 | 0,818233313 | 0,122546277 | 0,084739109 | 0,607719457 | 0,995094082 | 0,027661821 | 0,355803514 | 0,139151398 | 0,116448731 |
| 613 | 0,179819007 | 0,001153053 | 0,114080053 | 0,033045731 | 0,13359343 | 0,096996965 | 0,666546637 | 0,018608997 | 0,075936955 | 0,174189747 |
| 622 | 0,203534582 | 3,75138E-05 | 0,115321997 | 0,080998999 | 0,234020893 | 0,08059216 | 0,012495746 | 0,02549933 | 0,000652392 | 0,020743812 |
| 688 | 0,257477772 | 1,46277E-05 | 0,020145526 | 0,007967925 | 0,038997184 | 0,004274796 | 0,028743455 | 0,024893097 | 0,852991323 | 0,002399516 |
| 711 | 0,239111984 | 0,000820325 | 0,284906028 | 0,002082254 | 0,012106039 | 0,004404966 | 0,017227158 | 0,28311685 | 0,018608997 | 0,023124762 |
| 801 | 0,209561139 | 0,002507264 | 0,342770563 | 0,027140054 | 0,156987054 | 0,008572309 | 0,04936593 | 0,017007716 | 0,075936955 | 0,037488606 |
| 836 | 0,127611965 | 0,001193454 | 0,275717494 | 0,343568239 | 0,409033904 | 0,102062501 | 0,02549933 | 0,277032273 | 0,012203315 | 0,012429162 |
| 966 | 0,191524789 | 0,004466899 | 0,283284341 | 0,05656696 | 0,276305219 | 0,0105846 | 0,090993584 | 0,015191337 | 0,852991323 | 0,048668941 |
| 978 | 0,25945317 | 0,000652392 | 0,852991323 | 0,018608997 | 0,075936955 | 0,012203315 | 0,006442307 | 0,042042924 | 0,018608997 | 0,066821092 |
| 991 | 0,102183978 | 0,004623627 | 0,383186173 | 0,764025352 | 0,822086394 | 0,14223533 | 0,021228374 | 0,133528756 | 0,075936955 | 0,03668759 |
| 1088 | 0,243456358 | 0,000807277 | 0,692658802 | 0,038890552 | 0,105650498 | 0,030466733 | 0,012560106 | 0,006061404 | 0,012203315 | 0,020003059 |
| 1145 | 0,244510608 | 0,035312097 | 0,537645224 | 0,036060644 | 0,167428774 | 0,00095621 | 0,055152107 | 0,481971801 | 0,006442307 | 0,298678461 |
| 1154 | 0,273798338 | 4,69595E-05 | 0,110877331 | 0,003441326 | 0,020743812 | 0,002399516 | 0,023124762 | 2,56056E-05 | 0,035312097 | 1,38541E-05 |
| 1180 | 0,12116462 | 0,121068547 | 0,005726836 | 0,116448731 | 0,474385066 | 0,61236354 | 0,15149427 | 0,022682089 | 0,537645224 | 4,64316E-05 |
| 1181 | 0,09736916 | 0,070782517 | 0,006366891 | 0,174189747 | 0,447146338 | 0,968378001 | 0,378114047 | 0,017765124 | 0,036060644 | 0,001494012 |
| 1365 | 0,298678461 | 1,38541E-05 | 4,64316E-05 | 0,001494012 | 0,032930372 | 0,004566538 | 0,130646933 | 0,092584189 | 0,167428774 | 0,032930372 |
| 1372 | 0,237266641 | 4,46322E-06 | 0,037488606 | 0,012429162 | 0,048668941 | 0,066821092 | 0,03668759 | 0,020003059 | 0,133528756 | 0,004566538 |
| 1423 | 0,208497963 | 0,000709995 | 0,015191337 | 0,042042924 | 0,133528756 | 0,006061404 | 0,481971801 | 0,192858538 | 0,006061404 | 0,00095431 |

The manuscript offers significant insights into the lipidomic profiles associated with MASH and MASLD. However, addressing the suggested revisions will improve the manuscript's clarity and adherence to publishing standards. These revisions will also enhance the study’s overall methodological rigor and impact. I recommend that the manuscript undergoes revision before it is considered for publication.

**Reviewer #2**: In this study titled "Untargeted Lipidomic Analysis of Metabolic Dysfunction-Associated Steatohepatitis Associated with Morbid Obesity" by Bertran et al, authors have identified the key lipid changes associated with MASH. Although there are other lipidomics papers in similar lines, but this study has used a different cohort and presents some new findings. I think manuscript is straightforward and nicely written. My only suggestion is that authors must provide the complete lipidomics data set as the supplementary information.

**Response:** We have included the complete lipidomics dataset as the new Supporting Information (S1 Table).
