# Supplementary material for: Untargeted lipidomic analysis of metabolic dysfunction-associated steatohepatitis in women with morbid obesity
Source: PLoS One. 2025 Mar 4;20(3):e0318557. doi: 10.1371/journal.pone.0318557 (PMC11878907; doi:10.1371/journal.pone.0318557)
Supplement: Attachment — Submitted filename: Response to Reviewers_R2.docx [file pone.0318557.s008.docx]

Response to reviewers:

Reviewer #1

**Major Revisions**

- The statement "Finally, we have reported a panel of lipid metabolites that are discriminatory and specific to patients with MASH compared to SS patients, made up of increased levels of 9-HODE, some PC and PE, the LPI (16:0), and decreased levels of DG (36:0)." suggests strong discriminatory power of the identified metabolites. To support such claims, supervised analyses such as PLS-DA should be performed to calculate classification metrics, including accuracy, sensitivity, specificity, etc along with cross-validation. Similar studies, such as those outlined in https://www.mdpi.com/2073-4425/14/2/248, highlight the importance of validating panels with these metrics. In the conclusion, the statement "Thus, this panel of lipid metabolites could be used as a non-invasive diagnostic tool" should be revised to "might be used" unless robust classification results are presented.

**Response:** We appreciate your comment, so we have changed the sentence to be less conclusive.

- Detailed information on how missing values were handled should be included. Additionally, sharing scripts or pipelines for data preprocessing and analysis is critical for ensuring reproducibility.

**Response:** We included the method used in statistical analysis section. Our bioinformatics said that with all the information from the supporting information documents the reproducibility is ensured.

- Citations for all software and tools employed, such as Proteowizard’s MSConvert, RHermes, and XCMS, should be added. Suggested references include: https://pubs.acs.org/doi/10.1021/ac051437y , https://www.nature.com/articles/nbt.2377 and <https://www.nature.com/articles/s41592-021-01307-z>.

**Response:** We have included these references.

- PCA, while useful for exploratory purposes, focuses solely on variance and may not reveal discriminatory patterns between experimental groups. Supervised methods like PLS-DA, which leverage covariance to enhance group separation, are more suitable for this purpose. PLS-DA analysis should be conducted, and its results presented with proper classifications metrics to confirm discriminatory power. A Venn diagram based on supervised analysis results, highlighting metabolites specific to each comparison, would clarify findings further.

**Response**: We have already generated a PLS-DA with all the variables and another one with the significant ones, but it does not show separation between the groups. So, we believe the model is weak and we decided to perform a PCA (Figure 1) with the significant variables. In addition, we already included the Venn diagram as Figure 2.

- Perform functional enrichment analyses to provide deeper biological insights into the pathways and physiological implications of the identified metabolites. Tools like MetaboAnalyst (https://www.metaboanalyst.ca/) are recommended, and the tool should be cited if used.

**Response:** We are currently developing a manuscript for the functional analysis of the findings from the metabolomics, lipidomics, and proteomics study of this project. Therefore, we are working with that data for a new study and cannot include it in this one.

- Include database identifiers (e.g., HMDB, KEGG, PubChem, Wikipathways) for the detected metabolites. Specify the confidence levels of identification, following established standards for reporting, such as those outlined in <https://www.mdpi.com/2218-1989/10/1/8>.

**Response:** Given the lipid linkage, the lipidic metabolite name does not match its identifier, which is less specific. This is why we have included the exact formula of the lipid species in the supporting information S1 Table.

**Minor Revisions**

- The improved statistical notation in the main tables is commendable and enhances readability. Extending this notation to supplementary tables would ensure consistency. In supplementary tables where p = 0 is listed, it should be replaced with a threshold such as p < 0.0001.

**Response:** Accordingly, we have replaced it.

- Replace all occurrences of “triglycerides” with “triacylglycerols” and adopt the abbreviation TAG throughout the manuscript to align with international biochemistry guidelines.

**Response:** Accordingly, we have changed it.

- Ensure consistent use of abbreviations (e.g., PC for phosphatidylcholine) across the manuscript and supplementary materials.

**Response:** Done.

- Revise the sentence "Since we have found a common pattern of lipid metabolites in the comparisons between groups but have also observed that some metabolites are distinctive to the studied groups, we wanted to conduct a PCA to illustrate the distribution of these lipid metabolites among the groups (Fig. 1)." for clarity. Highlight that PCA reflects general variance and may not indicate group-specific patterns. Similarly, revise the discussion sentence "We could also mention that no exclusive lipid metabolite profile was shown in the PCA, so in this regard, the discriminative potential of the model is limited".

**Response:** Done.

- Enhance the Venn diagram by including the names or abbreviations of metabolites directly in the figure for better visualization.

**Response:** We attempted to include the variables in the same diagram, but it became overloaded, so we added a supplementary table in Supporting Information (S6 Table) with the overlapping variables between groups represented in the Venn diagram.

- Add a paragraph to the discussion summarizing the strengths and limitations of the study to provide a balanced perspective.

**Response:** Done.

- Include details about the commercial biochemical analyzer used for biochemical measurements, including the manufacturer and model.

**Response:** Done.

- Incorporate this information into Table 1: "In this sense, the percentage of patients in each group treated with lipid-lowering drugs was 19.4% of NL, 41.5% of SS, and 23.4% of MASH." with the statistical significance for clarity.

**Response:** Done.

- Provide all figures in a vector format (e.g., PDF or SVG) to ensure high-quality images suitable for publication.

**Response:** Done.

This manuscript is a valuable contribution to lipidomics research, and the revisions made thus far have strengthened its presentation. Addressing these additional suggestions will further improve the manuscript’s scientific rigor and impact.
